# ^18^F-FET-PET in Primary Hyperparathyroidism: A Pilot Study

**DOI:** 10.3390/diagnostics6030030

**Published:** 2016-08-17

**Authors:** Martin Krakauer, Andreas Kjaer, Finn N. Bennedbæk

**Affiliations:** 1 Department of Clinical Physiology and Nuclear Medicine, Gentofte Hospital, DK-2900 Hellerup, Denmark; 2 Department of Clinical Physiology, Nuclear Medicine & PET and Cluster for Molecular Imaging, Rigshospitalet and University of Copenhagen, DK-2100 Copenhagen, Denmark; andreas.kjaer@regionh.dk; 3 Department of Endocrinology, Herlev Hospital, DK-2730 Herlev, Denmark; finn.noe.bennedbaek@regionh.dk

**Keywords:** primary hyperparathyroidism, positron-emission tomography, (^18^F)fluoroethyl-l-tyrosine

## Abstract

Preoperative localisation of the diseased parathyroid gland(s) in primary hyperparathyroidism (PHP) is a prerequisite for subsequent minimally invasive surgery. Recently, as alternatives to conventional sestamibi parathyroid scintigraphy, the ^11^C-based positron emission tomography (PET) tracers methionine and choline have shown promise for this purpose. We evaluated the feasibility of using the ^18^F-based PET tracer fluoroethyl-l-tyrosine (FET), as the longer half-life of ^18^F makes it logistically more favourable. As a proof-of-concept study, we included two patients with PHP in which dual-isotope parathyroid subtraction single photon emission computed tomography had determined the exact location of the parathyroid adenoma. A dynamic FET PET/CT scan was performed with subsequent visual evaluation and calculation of target-to-background (TBR; parathyroid vs. thyroid). The maximum TBR in the two patients under study was achieved approximately 30 min after the injection of the tracer and was 1.5 and 1.7, respectively. This ratio was too small to allow for confident visualisation of the adenomas. FET PET/CT seems not feasible as a preoperative imaging modality in PHP.

## 1. Introduction

In primary hyperparathyroidism (PHP), excessive secretion of parathyroid hormone (PTH) from one or more hyperactive parathyroid glands causes elevated levels of blood Ca^2+^. In approximately 90% of cases, PHP is caused by a single parathyroid adenoma. The preferred treatment is surgical removal of the diseased gland(s). As the location of the parathyroid adenoma is variable, preoperative imaging is often performed in order to permit a minimally-invasive surgical approach. Current imaging modalities include ultrasonography, parathyroid scintigraphy, contrast-enhanced CT, and magnetic resonance imaging. Recently, positron emission tomography/CT (PET/CT) using the ^11^C-labelled amino acid derivative [^11^C-l-methyl]-methionine (MET) and the ^11^C-labelled vitamin-like choline has shown promise. However, the availability of the ^11^C-based tracers is limited because the short half-life of ^11^C (20 min) requires an on-site cyclotron. In our department, we routinely use the ^18^F-based tracer *O*-2-(^18^F)fluoroethyl-l-tyrosine (FET)—another amino acid derivative—for brain glioma imaging. The ^18^F-based PET tracers are much easier to handle and distribute due to the longer half-life of 2 h, and they also provide better spatial resolution due to a shorter positron range, thus making them more appealing for diagnostic use. We considered it likely that FET would perform comparably to MET, as both are amino acid derivatives.

Therefore, we carried out a “proof-of-concept” study in two patients, aiming to verify or refute the feasibility of further evaluation of FET-PET in the preoperative workup in PHP.

## 2. Results

**Figure 1:**
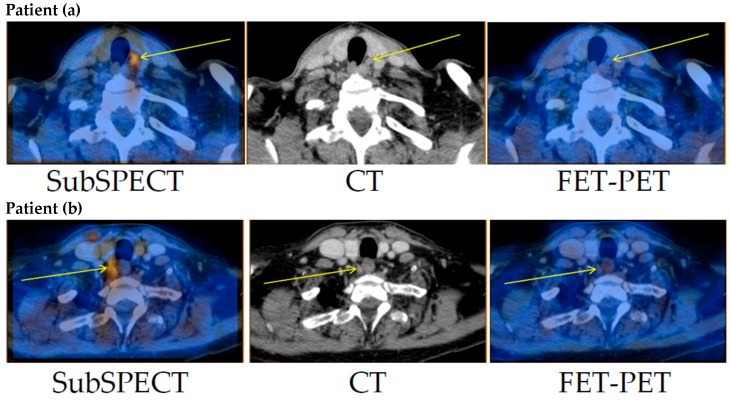
Tracer-uptake in the parathyroid adenomas in patients (**a**) and (**b**). **Left**: Subtraction (Tc-99m-sestamibi minus I-123)-SPECT/CT. **Middle**: CT. **Right**: FET-PET/CT. Thin arrows mark the surgically confirmed location of the parathyroid adenoma. FET: *O*-2-(^18^F)fluoroethyl-l-tyrosine; PET: positron emission tomography; SPECT/CT: single photon emission computed tomography/CT.

**Figure 2:**
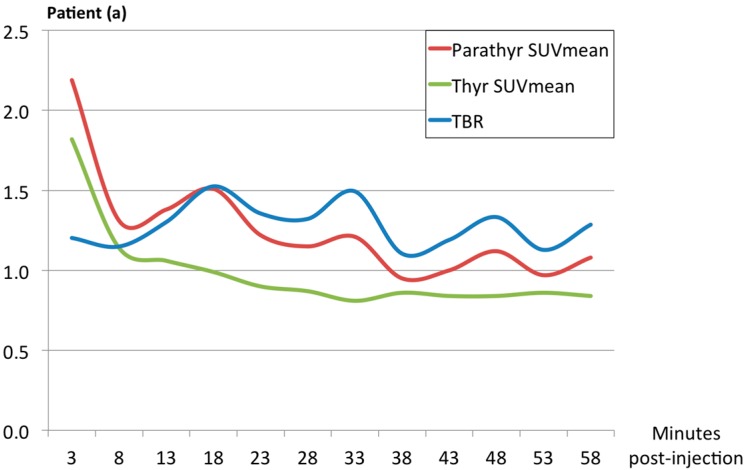

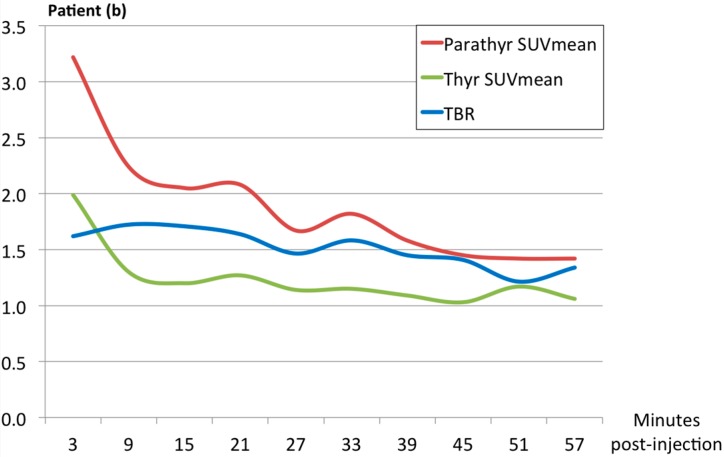
Region-of-interest based analysis of tracer-uptake in the first 60 min after tracer-injection in the parathyroid adenoma versus neighbouring tissue (thyroid) in patients (**a**) and (**b**). Target-to-background uptake value (TBR, blue) is the ratio between SUV_mean_ (mean standardized uptake value) in the parathyroid adenoma and SUVmean in the adjacent thyroid tissue.

Only faint FET uptake was detected in both thyroid tissue and the parathyroid adenomas (Figure 1). Excluding an obvious vascular phase during the first 10 min, TBR_max_ (for definition please refer to Materials and Methods) was reached between 30 and 35 min post-injection. TBR_max_ in patients (a) and (b) was 1.5 and 1.7, respectively. Thus, no clinically relevant differential uptake of FET could be detected in the parathyroid adenomas in either of the two patients (Figure 2).

## 3. Discussion

This study was purely of an exploratory nature aiming to assess the differential uptake of FET in parathyroid adenomas versus thyroid tissue in vivo. We found no significant differential FET uptake in either of the two patients studied.

As MET (another amino acid-derived tracer) shows high uptake in parathyroid adenomas, we had hypothesised that this would also be the case for FET. However, the mechanisms of cellular amino acid uptake are complex, especially with regard to radiolabelled amino acid derivatives such as FET and MET. Transmembrane amino acid uptake is facilitated by an intricate system of specific transport proteins, all with different affinities for the individual amino acids. Two major subgroups are the Na^+^-dependent and the Na^+^-independent transport systems, the latter including the l-type amino acid transporters (LAT1–4). The exact uptake mechanisms of FET and MET are not clear but it has been proposed that FET uptake primarily takes place via LAT2 [1], while MET uptake is primarily mediated by LAT1 [2]. Indeed, an antibody targeting the 4f2/SLC3A2 subunit of LAT1 has been shown to bind to and to affect parathyroid cells in culture [3], demonstrating the presence of a functional LAT1 receptor in parathyroid tissue. Conversely, functional LAT2 cell surface expression in parathyroid cells has not been reported, to our knowledge. These findings are consistent with the fact that we found no significant uptake of FET as opposed to that reported on MET in most parathyroid adenomas [4]. It could be argued that the two adenomas studied would also have been negative on MET. We cannot totally rule this out, as we did not perform MET-PET. However, since the sensitivity of MET-PET for surgically verified parathyroid adenomas has been found to be above 90%, we find it unlikely that both adenomas would have been negative on MET-PET.

## 4. Materials and Methods

This study was entirely exploratory. The primary aim was to study the in vivo uptake of FET in parathyroid adenomas.

The regional ethics committee for The Capital Region of Copenhagen approved the study (permission No. H-1-2011-101). After written and oral consent, patients were recruited from an ongoing study cohort evaluating various imaging modalities in PHP. As a tracer will only be appealing for clinical use if it provides increased sensitivity compared to the routinely used tracers, we tested the uptake of FET in two patients—(a) and (b)—with a positive sestamibi-single photon emission computed tomography/CT (SPECT/CT) performed with dual-isotope subtraction technique, which has a reported sensitivity of 93% [5]. The location of the parathyroid adenomas was subsequently surgically and histologically confirmed. The parathyroid adenomas from patients (a) and (b) weighed 0.83 and 0.22 g, respectively. Preoperative plasma-PTH was 163 and 142 ng/L, respectively.

A dynamic PET/low dose-CT scan of the neck in a single bed-position was initiated immediately after the injection of 200 MBq FET (Philips Gemini TOF PET/CT). Acquisition continued for 60 min in list-mode, and counts were re-binned into 5–6 min bins. The exact position of the parathyroid adenoma was determined by co-registering the subtraction SPECT/CT with the FET PET/CT. A region-of-interest based target-to-background uptake value (TBR) was calculated as the mean standardize uptake value (SUV_mean_) in the parathyroid adenoma divided by SUV_mean_ in the thyroid tissue in all bins. The bin with the highest TBR (TBR_max_) was determined.

## 5. Conclusions

We found no significant differential FET uptake in parathyroid adenomas, possibly due to lack of expression of specific transmembrane transporter molecules in parathyroid tissue. ^18^F-FET seems therefore not to be a feasible tracer for use in preoperative localisation imaging in PHP.
